# The continuum of attention dysfunction: Evidence from dynamic functional network connectivity analysis in neurotypical adolescents

**DOI:** 10.1371/journal.pone.0279260

**Published:** 2023-01-20

**Authors:** Halima Rafi, Farnaz Delavari, Nader Perroud, Mélodie Derome, Martin Debbané

**Affiliations:** 1 Faculty of Psychology and Educational Sciences, Developmental Clinical Psychology Research Unit, University of Geneva, Geneva, Switzerland; 2 Department of Psychiatry, Developmental Neuroimaging and Psychopathology Laboratory, University of Geneva, Geneva, Switzerland; 3 Medical Image Processing Lab, Institute of Bioengineering, EPFL, Lausanne, Switzerland; 4 Department of Psychiatry, Service of Psychiatric Specialties, University Hospitals of Geneva, Geneva, Switzerland; 5 Research Department of Clinical, Educational & Health Psychology, University College London, London, United Kingdom; Universidad Nacional Autonoma de Mexico, MEXICO

## Abstract

The question of whether attention-related disorders such as attention-deficit/hyperactivity disorder (ADHD) are best understood as clinical categories or as extreme ends of a spectrum is an ongoing debate. Assessing individuals with varying degrees of attention problems and utilizing novel methodologies to assess relationships between attention and brain activity may provide key information to support the spectrum hypothesis. We scanned 91 neurotypical adolescents during rest using functional magnetic resonance imaging. We conducted static and dynamic functional network connectivity (FNC) analysis and correlated findings to behavioral metrics of ADHD, attention problems, and impulsivity. We found that dynamic FNC analysis detects significant differences in large-scale neural connectivity as a function of individual differences in attention and impulsivity that are obscured in static analysis. We show ADHD manifestations and attention problems are associated with diminished Salience Network-centered FNC and that ADHD manifestations and impulsivity are associated with prolonged periods of dynamically hyperconnected states. Importantly, our meta-state analysis results reveal a relationship between ADHD manifestations and exhibiting variable and volatile dynamic behavior such as changing meta-states more often and traveling over a greater dynamic range. These findings in non-clinical adolescents provide support for the continuum model of attention disorders.

## Introduction

Attention and impulsivity are continuously varying psychological processes influenced by individual differences in genetics, personality, as well as cognitive and affective processing [1,2]. Clinical evaluations of attention and impulsivity are designed to tease apart variation at the extreme end of said continuum, which is typically when attention disorders such as attention deficit hyperactivity disorder (ADHD) are diagnosed [3]. Adopting a strictly categorical lens comes with drawbacks: it ignores that typically developing (TD) individuals show important variation in attention and impulse-control that can lead to significant functional challenges [4]. A categorically-truncated view of population variance decreases data reliability, validity and statistical power, making the identification of biomarkers for psychiatric conditions less probable [5,6]. In addition, attention disorders such as ADHD have high comorbidity rates with other psychiatric disorders, such as oppositional defiant disorders and anxiety disorders [7,8]. One way to side-step these confounding problems is to examine the variation of attention and impulsivity, and their associated neural mechanisms, within TD individuals. Similar to other fields of developmental psychopathology [9,10], the present study adopts a continuum approach towards attention-disorder related problems in order to contribute meaningful insights into the neural signature of attention disorders.

Spatially distributed and functionally linked brain networks can be reliably identified during adolescence [11]. Functional magnetic resonance imaging (fMRI) studies have increasingly used the triple network theory framework to research how complex interactions between these networks influence psychological processes in clinical populations [12–14]. The triple network theory posits that atypical activity of three large-scale networks [15], the default mode network (DMN), executive control network (ECN) and salience network (SN), underlies neurodevelopmental disorders. The DMN is arguably the most well-researched neural network, and is a task-negative network associated with mind-wandering and social cognition (for reviews, see [16,17]). It can be divided into the dorsal DMN (dDMN), including the anterior cingulate/medial prefrontal cortices, and the ventral DMN (vDMN), including the posterior cingulate cortex (PCC) and precuneus [17–19]. Attentional difficulties such as temporary lapses of attention [20] have long been associated with the DMN, so much so that ADHD was thought to be a disorder of the DMN [21–23]. This claim was partly supported by research showing ADHD populations have atypical functional network connectivity (FNC) within the DMN [23,24], difficulty suppressing DMN activation when switching from rest to task-focused cognitive activity [22], as well as abnormal functional connectivity between the DMN and cingulo-opercular and occipital regions [25,26]. However, current literature is inconsistent, with studies showing attention-related DMN hypo-connectivity [23,24,27], hyper-connectivity [28,29] as well as a combination of both [30]. The remaining networks of the triple network theory, the ECN and SN, are both task-positive networks involved in higher-order cognitive control; the ECN encompasses the dorsal lateral prefrontal cortex and helps integrate sensory and memory information, as well as regulate cognition, behavior and executive functions [31,32]. The SN is anchored in the insular cortex and the anterior cingulate cortex (ACC), and responds to external events that are behaviorally salient [33]. Importantly, both networks are distinct from the dorsal attention network, which is comprised of the frontal eye fields and the intraparietal sulcus [34].

Patterns of FNC between the DMN, ECN and SN at rest overlap with patterns seen during goal-directed behaviors [35–37], suggesting this triad of networks is important for regulating attention, cognition and affect [38]. In particular, the SN is thought to allow for flexible cognitive control by regulating interactions between the DMN and ECN [13,39]. Subsequently, the failure to regulate DMN-ECN interactions is hypothesized to underlie task interference and downstream attention problems [40]. Populations characterized by strong attention difficulties, such as ADHD, consistently show deficits in engaging and disengaging the SN, ECN and DMN compared to TD populations (for a meta-analysis, see [41]). Recent literature increasingly suggests that FNC centered around the SN, rather than the DMN, may represent a neural signature of childhood attention disorder symptoms [33,39,42].

Dynamic FNC analysis is a systems neuroscience approach to quantifying how neural networks interact over time [43,44]. This analysis identifies different dynamic states that represents a distinct, cross-network activation pattern that participants oscillate in and out of over time [43]. State-based metrics can be calculated from dynamic FNC analysis, such as mean dwell time, which is the time spent in a certain state before switching to another, fraction time, which reflects time spent in one state relative to the entire scan time and the number of transitions, which indicates how often a participant changed states [43]. Importantly, a novel measure of dynamism also derived from dynamic FNC analysis is meta-state analysis, which adopts a complex statistical approach and finer temporal resolution to calculate summary measures of brain dynamism. As compared to dynamic FNC analysis, meta-state analysis is better able to capture the dynamic fluidity and range of large-scale neural connectivity [45].

The first study to adopt a dynamic approach to FNC in ADHD reported diminished connectivity between the SN-DMN and the SN-ECN in young children with ADHD compared to controls, which correlated with the severity of inattention symptoms [13]. It also revealed that children with ADHD had less persistent brain states that lasted for shorter periods of time and fewer cross-network interactions as compared to controls. This hallmark study contributed to accumulating evidence that diminished SN-centered FNC plays a critical role in attention problems in children. It remains unknown whether this pattern of FNC continues to be associated with attention and impulse-control problems in later developmental stages such as adolescence. Adolescence is a formative developmental period with a unique mix of pubertal, social and academic changes that influence neural and psychological maturation [46]. Despite their overall stability, large-scale brain networks undergo subtle reorganization during adolescence (for review see [47]) at the same time that attention-related functional impairments are often diagnosed. Given its unique window of analysis, dynamic FNC analysis may represent a key methodology for exploring the properties of time varying neural connectivity and its relationship to attention problems and impulsivity during adolescence.

Despite a long-standing debate on whether ADHD should be reclassified as a spectrum disorder, it is unknown whether ADHD symptomology correlates with FNC patterns in TD adolescents. To assess this, we scanned TD adolescents during rest and assessed both static and dynamic patterns of FNC. We followed a precedent set by a recent dynamic FNC study with a dimensional approach to clinical disorders [48] and assessed how FNC patterns related to ADHD manifestations, attention problems and impulsivity. We hypothesized that dynamic FNC would allow for the detection of activation patterns obscured in time-averaged FNC, namely that TD adolescents with greater attentional difficulties and impulsivity would show more variability in cross-network interactions as assessed by dynamic state metrics as well as meta-state features. In line with previous research in clinical populations [13], we expected a continuation of diminished SN-centered connectivity in adolescents with higher up on the spectrum of attention-disorder related impairment.

## Materials and methods

### Participants

We recruited 91 TD adolescents between the ages of 12 and 17 years (mean age = 15.4 ± 1.7 years, 42 females) from Geneva, Switzerland, and surrounding regions. Inclusion criteria included no previous psychiatric diagnosis, epilepsy, or neurological disorders, no intellectual impairments (based on the Cubes and vocabulary subtests of the Wechsler Scales of Intelligence for children (WISC-IV [49])) and normal or corrected-to-normal vision. Participants received financial compensation, and written consent was obtained from their parents or legal guardians under protocols approved by the local ethical commission (Commission Centrale d’éthique de la Recherche des Hôpitaux Universitaires de Genève) and in accordance with the Declaration of Helsinki. From the original sample, 9 participants were excluded for excessive movement, defined as a maximum displacement (rotation or translation) of more than 3.0 mm during the fMRI scan. An additional 2 participants were excluded for incomplete behavioral data, resulting in a final sample of 80 adolescents (average age = 15.6 ± 1.6 years, 38 females). There were no behavioral differences in terms of attention nor impulsivity between the 80 included participants and 9 excluded participants (S1 Table). Demographic and behavioral data for included participants can be found in Table 1.

**Table 1:** All participants demographic and behavioral data. Demographic data, means and standard deviations for all behavioral measures of interest for included and excluded participants.

	Group	N	N_Females_	Mean	Std. Deviation
**Age**	Included	80	38	15.64	1.63
	Excluded	9	6	15.51	1.62
**IQ (WISC-IV, Cubes Standardized Score)**	Included	80	38	10.74	3.23
	Excluded	9	6	9.89	3.69
**YSR Attention Problems**	Included	80	38	57.19	6.85
	Excluded	9	6	61.27	12.17
**YSR ADHD**	Included	80	38	57.00	6.67
	Excluded	9	6	58.55	4.69
**YSR Internalizing**	Included	80	38	51.93	9.92
	Excluded	9	6	53.81	12.56
**YSR Externalizing**	Included	80	38	56.23	8.84
	Excluded	9	6	58.09	9.43
**UPPS Urgency**	Included	80	38	2.46	0.66
	Excluded	9	4	2.36	0.43
**UPPS Lack of Premeditation**	Included	80	38	2.21	0.63
	Excluded	9	4	2.38	0.75
**UPPS Lack of Perseverance**	Included	80	38	2.04	0.64
	Excluded	9	4	2.20	0.85
**UPPS Sensation Seeking**	Included	80	38	2.82	0.64
	Excluded	9	4	2.90	0.80

### Questionnaires

Participants completed the Achenbach Youth Self Report [50] (YSR), which assesses behavioral problems in the previous 6 months using a 3-point scale (0 = not true to 2 = very true). Subscales include attention problems, attention deficit/hyperactivity, somatic complaints, social problems, thoughts problems, anxiety/depression, withdrawal/depression, rule-breaking behavior and aggressive behavior. The attention deficit/hyperactivity subscale is one of the YSR’s DSM-oriented subscales and while it was not designed to be a perfect equivalent of the DSM’s ADHD criteria, it has nonetheless been found to be an accurate screener for ADHD. Due to the present study’s focus on attentional difficulties, the two most relevant YSR subscales were selected from a version of the YSR validated for French-speakers [51], namely attention problems and attention deficit/hyperactivity. Participants also completed the Urgency-Premeditation-Perseverance-Sensation Seeking (UPPS) Impulsive Behavior Scale [52] a self-report questionnaire measuring four facets of impulsivity. The four facets are sensation seeking, lack of deliberation, lack of perseverance and urgency, all of which have been validated in diverse populations including TD children and adolescents [53–55], TD adults [56,57] as well as various clinical populations [58,59]. Given the large age range of our sample, we controlled for developmental effects by correlating all behavioral measures with age. No significant results were found.

### Data acquisition

Anatomical and functional resting-state imaging data were acquired on a 3T Siemens Trio scanner. The T1-weighted sequence was collected with a 3D volumetric dimension using the following parameters: TR = 2500 ms, TE = 3 ms, flip angle = 8°, acquisition matrix = 256 x 256, field of view = 22 cm, slice thickness = 1.1 mm, 192 slices. An 8-minute resting state fMRI sequence was used during which subjects were asked to fixate their eyes on a white cross shown on a black screen, let their thoughts wander and refrain from falling asleep. We verified that participants kept their open during the scan using an in-scanner eye-monitor and, after the completion of the resting state scan, all participants were asked to describe their experience of the resting state scan and to disclose if they had fallen asleep at any point during the scan. Head movement was minimized with a vacuum cushion constraint. 200 BOLD images were acquired using the following parameters: TR = 2400 ms, TE = 30 ms, 38 axial slices, slice thickness = 3.2 mm, flip angle = 85°, acquisition matrix = 94 x 128, field of view = 96 x 128.

### Data analysis

#### fMRI data preprocessing

For each participant, the first 10 functional volumes were discarded to control for equilibration effects of the T1 signal and functional volumes were manually reoriented to place the origin at the anterior commissure. fMRI data were preprocessed in a standardized manner using Data Processing Assistant for Resting-State fMRI (DPARSF) software (http://rfmri.org/DPARSF) [52] implemented using MATLAB [53]. DPARSF is based on Statistical Parametric Mapping (SPM12, http://www.fil.ion.ucl.ac.uk/spm) and the Resting-State fMRI Data Analysis Toolkit (http://www.restfmri.net) [54]. More specifically, data were realigned, slice-timing corrected, co-registered to respective structural images of each subject and segmented. Six rigid body motion parameters, white matter and cerebrospinal fluid signal were regressed out. Images were normalized using Diffeomorphic Anatomical Registration using Exponential Lie algebra (DARTEL) to create a population-specific template, which was then spatially normalized to standard stereotaxic space based on the Montreal Neurological Institute (MNI) coordinate system. Spatial smoothing was applied using an isotropic Gaussian smoothing kernel with a full width at half maximum (FWHM) of 5mm to decrease noise and data was filtered using a temporal band-pass (0.01–0.08 Hz) to reduce the effect of low-frequency noise such as respiration, and high-frequency noise such as cardiac activity.

#### Group ICA analysis

**Fig 1:**
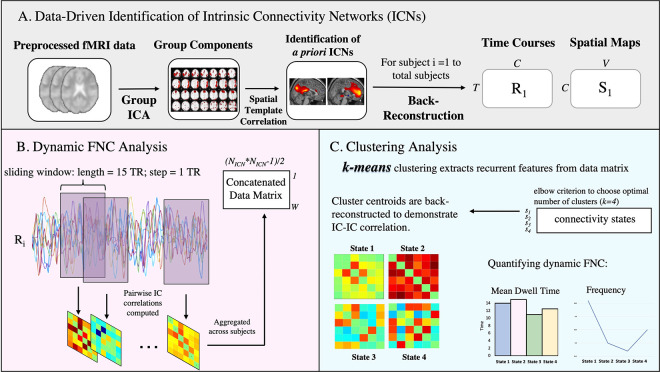
Illustration of major data analysis steps. A) Group ICA analysis was run on preprocessed subject data, resulting in 29 independent components (*C* = 29), 6 of which were identified as ICs in the DMN, ECN and SN. GICA1 back-reconstruction was used to estimate the time courses (*R*_*i*_) and spatial maps (*S*_*i*_) for each subject. B) The sliding window approach was used to estimate dynamic FNC as the series of correlation matrices from windowed portions (*W*) of *R*_*i*_, resulting in a concatenated data matrix of all IC-IC paired correlation values over time. C) *K*-means was performed on the concatenated data matrix as outlined in Allen et al., 2014. The optimal cluster number was k = 4 and each windowed FNC was assigned to a cluster. Each cluster centroid (also known as state) is represented by a correlation matrix. Clustering analysis allows for quantifying dynamic FNC through measures such as Mean Dwell Time and Fraction Time. This figure was adapted from El-Baba and colleagues [60].

Preprocessed images were analyzed using the Group ICA of fMRI Toolbox (GIFT) software package (v4.0b; Medical Image Analysis Lab, University of New Mexico; http://icatb.sourceforge.net/groupica.htm). 29 independent components (ICs) were assumed, based on dimension estimation with minimum description length of the data. Group ICA was performed on fMRI data in three steps: data reduction, ICA, and back reconstruction. In the data reduction step, the data of each participant was reduced using principal component analysis (PCA). The data was then concatenated into a group and reduced again using PCA analysis. In the ICA step, the data underwent IC estimation. IC estimations were stabilized by repeating the ICA algorithm 20 times using ICASSO (http://research.ics.tkk.fi/ica/icasso). The Iq index from ICASSO was used to validate IC decomposition stability and only stable components with an index Iq value greater than 0.8 were retained. Finally, GIFT uses GICA1 back‐reconstruction to create subject‐specific time courses and spatial maps. For a schematic representation, please refer to Fig 1.

#### Identification of resting state networks

Valid networks of interest were identified by visual inspection and confirmed by spatial correlations with publicly-available functional network templates [61] (http://findlab.stanford.edu/functional_ROIs.html). For each network of the triple network theory, the IC with the largest correlation coefficient was chosen (S3 Table). In accordance with our hypothesis, 6 ICs were chosen (ICs 22, 13, 7, 11, 14 & 27) that corresponded to the following 6 networks: dorsal DMN (dDMN), posterior ventral DMN (pvDMN), posterior dorsal DMN (pdDMN), right ECN (rECN), left ECN (lECN) and the SN (for a schematic representation, please refer to Fig 1). Additional networks such as the basal ganglia, auditory, language, sensorimotor, visuo-spatial, and visual were also identified but not used in further analysis because they were outside the scope of the triple network theory.

#### Static FNC analysis

Static FNC analysis was conducted using the MANCOVAN toolbox in GIFT (v4.0b; Medical Image Analysis Lab, University of New Mexico; http://icatb.sourceforge.net/groupica.htm). Analyses followed step-by-step procedures described in previous studies [62]: first, each subject’s time course was detrended, de-spiked and filtered using a fifth-order Butterworth low-pass filter with a cutoff frequency of 0.15 Hz. Age, sex, frame-wise displacement and each subject’s motion parameters (rp*txt) were included as nuisance covariates [12]. For each participant, a correlation map was produced by computing the correlation coefficient *r* between the time series of each pair of ICs (the ICA algorithm assumes that the time courses of cortical areas within one component are synchronous [63]). Given that we had six networks of interest, a total of fifteen different pair-wise combinations of inter- and intra-network connectivity were obtained.

#### Static FNC statistical analysis

Before running statistical analysis on static FNC results, we transformed *r* values into z-scores using Fisher’s transformation. We verified the data was normally distributed and used Pearson correlations to correlate each participant’s measure of functional network connectivity with behavioral scores of attention problems and ADHD manifestations (as measured by the YSR subscale [50]) and impulsivity (as measured by the UPPS Impulsive Behavior Scale [52,64]. Correlation results were corrected for multiple comparisons for univariate analyses (for both number of IC pairs and number of behavioral questionnaires) using the false discovery rate (FDR; *p*<0.05). All statistical analyses were performed in RStudio (http://www.rstudio.com/).

#### Dynamic FNC analysis

We conducted dynamic FNC analysis using the tapered sliding window approach [43,44] to identify distinct, time-varying patterns of FNC. Critically, we chose to model our analysis based off of parameters used in recent dynamic FNC studies [12,43,65], including one that also adopted a dimensional approach towards clinical disorders [48]. In accordance with said studies, a rectangular window (width 15 TRs or 36 seconds) was convolved with a Gaussian of sigma 3 TRs and slid in steps of 1 TR across concatenated time courses, resulting in 160 time-windowed domains per subject. A separate FNC matrix was computed as the pairwise correlation between networks of interest (6 network x 6 networks) for each of the 160 time-windowed domains (per subject). In total, the dynamic FNC data was made up of 12800 windowed FNC matrices (80 participants * 160 windowed FNC). These windowed FNCs capture the changes in covariance between our 15 networks during the duration of the scan. Other important statistical measures included the graphical LASSO algorithm [66], which was used to improve the estimation of correlations among time-courses with short time domains, as well as a penalty on the L1 norm of the precision matrix to increase sparsity. The regularization parameter was optimized for each subject by evaluating the log-likelihood of unseen data (subject’s covariance matrices) in a cross-validation framework. For a schematic representation, please refer to Fig 1.

#### Clustering analysis

K-means clustering was applied to windowed FNCs for both dynamic FNC and meta-state analysis. For dynamic FNC, we used the k-means algorithm with the L1 distance (Manhattan distance) to run clustering analysis [48], a validated approach used to identify which FNC states had most commonly occurred during rest [43,67]. K-means was run on all subjects’ dynamic FNC data with the number of clusters ranging from two to eight. Parameters for k-means included 10 cycles of clustering with a maximum of 200 iterations for reaching a solution. The elbow criterion was then applied to the resulting cluster index to estimate the optimal number of clusters, which was K = 4. The four clusters, also referred to as FNC states, described the four connectivity patterns that subjects move between over time. Given that every subject’s trajectory between the four FNC states was different, it is important to note that not every subject entered every state. Based on the dynamic FNC states, three metrics characterizing each participant’s dynamic behavior during the scan were calculated. These behavioral metrics include mean dwell time (MDT), which is the time spent in a certain state before switching to another one, fraction time (FT) which is the time spent in one state relative to the entire scan time and the number of transitions (NT), which represents how often participants changes between different dynamic states.

#### Meta-state analysis

The difference between dynamic FNC analysis and meta-state analysis begins after the sliding window has dissected data into windowed FNC matrices. Rather than assigning each windowed FNC to one dynamic state as done in dynamic FNC analysis, meta-state analysis models each windowed FNC as weighted sums of maximally independent connectivity patterns [45]. These connectivity patterns are then discretized using quartile discretization. The discretized connectivity pattern distance vectors are referred to as meta-states [14]. Four indices of connectivity dynamism can be calculated from meta-states, namely i) the number of distinct meta-states the subjects occupied during their scans (meta-state number); ii) the number of times that subjects switched from one meta-state to another (meta-state changes); iii) the largest distance between two meta-states that subjects occupied (meta-state span); and iv) the total distance traveled by each subject through the state space (meta-state total distance).

#### Dynamic FNC statistical analysis

Dynamic connectivity was assessed through three separate analyses, namely dynamic FNC, state-based metrics, and meta-state metrics. Statistical tests between each of these analyses and behaviors of interest (Attention Problems and ADHD Manifestations, as measured by the YSR [50], and impulsivity, as measured by the UPPS Impulsivity Scale [52,64]), were computed using RStudio (http://www.rstudio.com/).

Dynamic FNC analysis output a matrix for each dynamic state, which consisted of correlation coefficients (r values) for each of the 15 IC-IC pairs, for each participant who entered that state. We stabilized variance by using the Fisher transformation to convert r values into z-scores. We then conducted Pearson correlations between the 15 IC-IC pairs and our 6 behavioral metrics of interest, for each of the four dynamic states. The results of this correlation analysis were corrected for multiple comparisons (360 comparisons) for univariate analyses using the false discovery rate (FDR; p<0.05).

We obtained 3 state-based metrics for each participant for each state (MDT, FT, and NT). We made one matrix with each participant’s state-based metrics for all 4 states (MDT state 1, MDT state 2 etc.) and behavioral data and ran Pearson correlations and corrected for multiple comparisons (1440 comparisons) for univariate analyses (FDR; p<0.05).

We obtained 4 meta-analysis metrics (meta-state number, meta-state changes, meta-state span & meta-state total distance). We made one matrix with each participant’s meta-state data and behavioral results ran Pearson correlations. The results of this correlation analysis were corrected for multiple comparisons (1920 comparisons) for univariate analyses (FDR; p<0.05).

## Results

### Static FNC results & behavioral correlations

During rest, the three ICs of the DMN (vDMN, pdDMN, and pvDMN) were most strongly correlated with themselves, as were the two ICs of the ECN (rECN and lECN) (Supplementary Materials, S1 Fig). The posterior components of the DMN had weak positive correlations with both the SN and rECN. The ECN and SN were neither correlated nor anti-correlated with each other. No correlations between static FNC and behavioral measures survived multiple comparison correction.

### Dynamic FNC results

**Fig 2:**
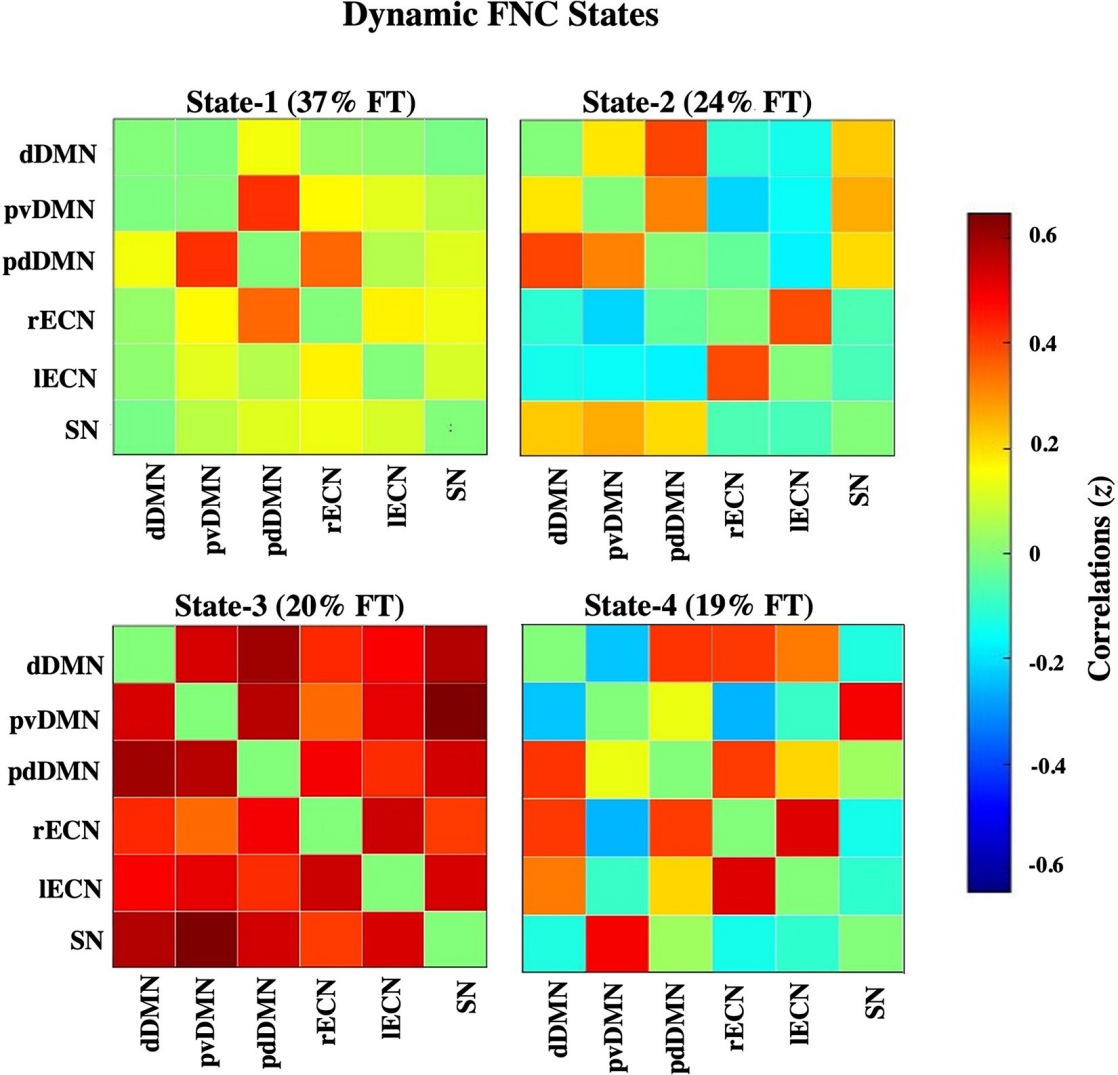
Dynamic FNC results. Correlation matrices showing the fraction time and pattern of cross-network functional connectivity (represented using z-scores) of each of the four dynamic FNC states. 74 participants entered State-1, 66 participants entered State-2, 54 participants entered State-3, and 50 participants entered State-4. dDMN = dorsal default mode network; pvDMN = posterior ventral default mode network; pdDMN = posterior dorsal default mode network; rECN = right executive control network; lECN = left executive control network; SN = salience network.

Dynamic FNC analysis revealed that time-varying FNC in our sample could be represented in 4 distinct states (Fig 2) rather than a single, static state [48]. State-1 was the most frequent, with an average fraction time of 37%, and represented a dynamic state in which posterior DMN and ECN were harmonically connected and segregated from the SN. ICs in State-1 shared weak positive correlations with each other, except for the posterior DMN, which was strongly correlated within itself (pdDMN-pvDMN) and with the right ECN (pdDMN-rECN). 74 out of the 80 participants spent time State-1. State-2 was the second most frequent state with an average fraction time of 24%. It displayed patterns of resting state activation typically seen in time-averaged FNC, with strong positive correlations within nodes of the DMN and the ECN, respectively, anti-correlations between the DMN-ECN and moderately strong correlations between the DMN-SN. 66 out of 80 participants spent time in State-2. State-3 had an average fraction time of 20% and showed positive connectivity between all networks. 54 out of 80 participants spend time in State-3. Finally, State-4 had an average fraction time of 19% and can be defined by a strong and isolated synchronization between the pvDMN-SN; both the pvDMN and SN were either not correlated or anti-corrected with the remaining ICs namely the dDMN, pdDMN and ECN, which were, in turn, positively correlated amongst themselves correlated with each other. 50 out of 80 participants spend time in State-4. Importantly, to confirm our results were not majorly driven by motion difference among the participants. we calculated Pearson correlations between the rate of occurrence of each dynamic state and mean framewise displacement of our participants. We found no significant correlation (S3 Table).

### Associations between dynamic FNC, state-based metrics and behavior

**Fig 3:**
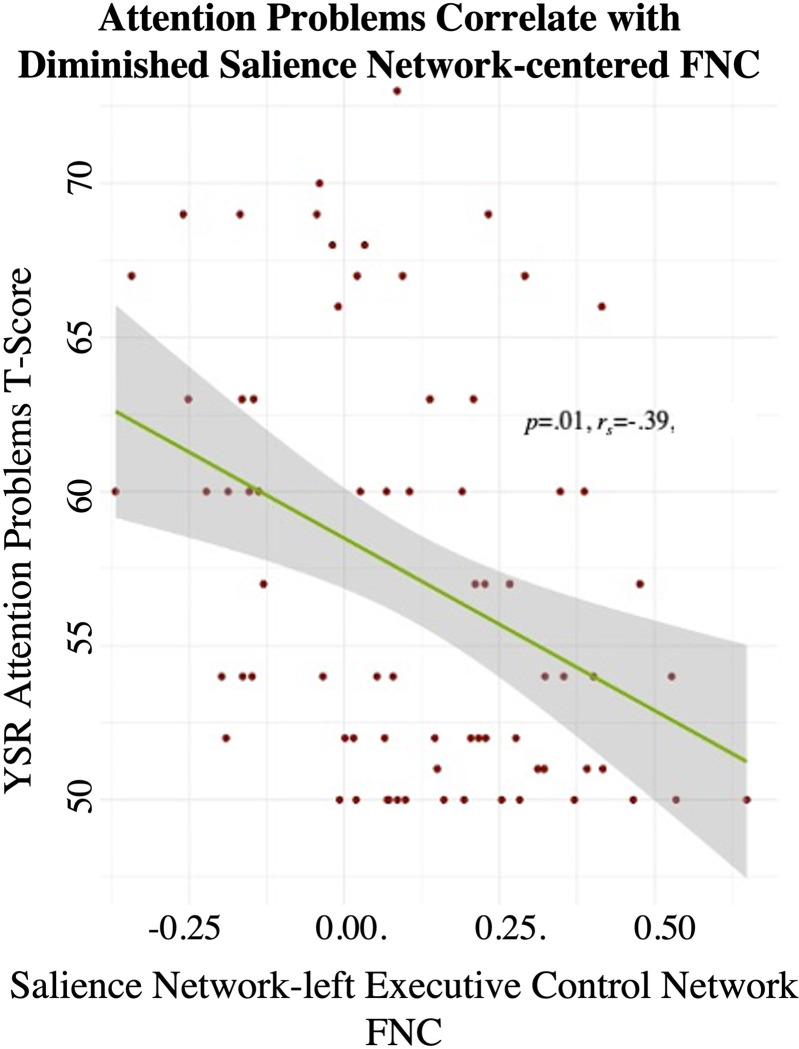
Association between FNC in dynamic state-1 and attention problems. Pearson correlations revealed that participants’ T-Scores for Attention Problems (as measured by the YSR) correlated with diminished functional connectivity between the Salience Network and the left Executive Control Network during Dynamic State-1 (No other correlations survived correction for multiple comparisons (FDR; p<0.05).

To assess relationships between dynamic FNC and behavior, we computed Pearson correlations between each dynamic state (State-1, State-2, State-3 and State-4) and behaviors of interest, namely, Attention Problems & Manifestations of ADHD (as measured by the YSR [50]) and impulsivity (as measured by the UPPS Impulsivity Scale [52,64]. Results revealed that, unlike the static connectome, dynamic connectivity patterns did correlate with variations seen in behavior. This was illustrated by two behavioral scores that correlated with diminished SN-lECN functional connectivity during State-1: first, Manifestations of ADHD correlated with diminished FNC between the SN and the lECN (*p* = .02, *r* = -.38) and second, Attention Problems also correlated with diminished FNC between the SN and the lECN (*p* = .01, *r* = -.39) (Fig 3). No other correlations survived correction for multiple comparisons (FDR; p<0.05).

We also assessed relationships between state-based metrics and behavior. Results revealed that mean dwell time in State-3 correlated with both ADHD manifestations, as measured by the YSR [50] (*p* = .03, *r* = .30), and impulsivity, as assessed by the Lack of Perseverance subscale of the UPPS-S [52,64] (*p* = .02, *r* = .33). No other correlations survived correction for multiple comparisons (FDR; p<0.05).

### Associations between meta-states and behavior

Meta-state analysis revealed positive associations between ADHD manifestations, as measured by the YSR [50] and the number of times participants changed meta-states (*p* = .04, *r* = .24) as well as the total distance (*p* < .01, *r* = .35). No other correlations survived correction for multiple comparisons (FDR; p<0.05).

## Discussion

This study assessed whether dynamic FNC analysis could detect meaningful relationships between large-scale brain networks and ADHD manifestations, attention problems and impulsivity in TD adolescents, that were obscured in static FNC. Only one study to date has used the triple-network model of cognitive control to test the hypothesis that SN-centered interactions are impaired in ADHD [13] and no study has assessed how time-varying interactions relate to attention disorder-related symptoms in TD adolescents.

Overall, our results suggest that dynamic FNC is a sensitive approach to underpinning fine alterations in large-scale neural circuits and their behavioral correlates in non-clinical populations. We found no significant relationship between behaviors associated with attention disorders and static FNC patterns in TD adolescents. Static FNC analysis is a well-known methodology that has been linked to behaviors associated with clinically diagnosed attention disorders [13], the present lack of findings suggests our sample size of TD adolescents may not have been adequately large enough to detect what may be relatively small effects. To assess whether a different approach would yield results, we conducted dynamic FNC analysis which revealed 1) that network interactions between the DMN, ECN and SN in our sample could be optimally represented through four dynamic states (Fig 2) and 2) that there is enough variation within TD populations to allow for detection of relationships between large-scale networks and behaviors associated with attention disorders.

Most participants entered and spent the most time in State-1, which showed strong connectivity within the posterior DMN and between the DMN-ECN along with a complete absence of SN-centered connectivity. Remarkably, within this dynamic state, we were able to observe lower FNC between the SN-ECN in participants with higher scores for attention problems. This lends additional evidence that SN-centered connectivity contributes to clinical manifestations of attentional disorders (see recent review [68]). The anterior insula, a hub within the SN [33], is believed to play a crucial role in mediating switching between the ECN and the DMN. The SN-ECN connectivity specifically is thought to signify the detection of salient stimuli by the anterior insula and subsequent signaling to the ECN to recruit recourses necessary for attentionally demanding tasks [33,69]. Previous research has linked aberrant activation the SN, including the anterior insula, to ADHD [70]. In the present study, the similarity of diminished SN-centered connectivity to behavioral measures of both ADHD and Attention Problems may suggest that the former is being driven by the latter. Moreover, the fact that we can observe attention-related impairments in the SN-ECN in TD populations suggests that this circuit may be most relevant to attention problems in ADHD and could potentially be influenced by aberrant structural connectivity between the SN-ECN. We also find results relevant to impulse-control/hyperactivity seen in ADHD: in our sample, participants higher up on the spectrum of both ADHD manifestations and impulsivity problems spent longer in the hyperconnected State-3 before switching to another state. This finding uncovered by dynamic FNC analysis suggests two things: first, that while all TD adolescents may enter and leave hyperconnected states during rest, such states are more stable in adolescents with increased ADHD manifestations. Second, given that State-3’s mean dwell time also correlated with increased impulsivity, having prolonged periods of time where large-scale neural networks are hyperconnected to each other may be specific to the hyperactive manifestations of ADHD.

Critically, we present evidence of enhanced global dynamic activity over an extended dynamic range, reflected by increasingly volatile meta-states that change more often and travel a greater total distance, in adolescents with increased ADHD manifestations. Meta-state analysis aims to account for as much information within each windowed FNC as possible by creating a vector representing the distance of each windowed FNC to each dynamic state [45] and, in doing so, circumvents issues with the more conservative dynamic state-based metrics, which assign every windowed FNC to its most highly correlated state. If dynamic FNC analysis is viewed as a more comprehensive, process-focused extension of static FNC analysis, meta-state analysis can be viewed as a similar continuation of dynamic FNC. Assessing the number of unique meta-states and other meta-state metrics may allow for the detection of subtle relationships between attention and FNC that state-based dynamic metrics, which are comparatively more conservative, only capture in clinical samples. Here, we present results indicating the continuation of that same finding in TD adolescents. In other words, previous dynamic FNC studies have shown that children with ADHD oscillate between a greater number of dynamic FNC states and have more variable network interactions than TD children [13]. In the present study, we present evidence that this phenomenon is not restricted to categorically defined, clinical ADHD, but that non-clinical adolescents higher up on the *spectrum* of attention dysfunction also follow volatile patterns of changing meta-states more often and trending towards oscillating between a greater number of meta-states than adolescents lower down on the same continuum. We also show that the total distance traveled by each subject through the state space (the sum of the L1 distances between successive meta-states, i.e., meta-state total distance) was greater for participants higher up on the attention problem continuum. When taken with our previous findings, we show that as ADHD manifestations increase, adolescents tended to spend more time in a state of hyperconnectivity while also traveling over an increased dynamic range, once again supporting the pattern of increased volatility associated with attention disorders.

We acknowledge the present study has several limitations. Future studies should aim to include behavioral instruments measuring attention problems and impulsivity that are less dependent on participant self-report. This is particularly the case for ADHD populations [71], for whom it is important to include performance tasks sensitive to attentional disturbances such as the continuous performance test (CPT) [72]. Future studies should also aim to include measures of hyperactivity, a core symptom in attention-related disorders, especially during adolescence. In terms of task validity, we instructed participants to let their mind wander without falling asleep during the scan. We used an in-scanner eye monitor to ensure they did not close their eyes but did not include questionnaires about thoughts or cognitions afterwards to get an indication of their scan experience, which could have influences static and dynamic connectivity [73]. It is important to underline that we did not directly compare static to dynamic FNC and therefore cannot state that dynamic FNC is better suited to uncovering trends in non-clinical populations. In terms of the applicability of the present results, it is of high importance to repeat these analyses in populations with and without clinical ADHD and to do so longitudinally. Pursuing such analyses will bring further insights into the clinical states of ADHD and its presentations, and into common clinical and theoretical challenges such as comorbidity in ADHD and functional outcomes along development. While out of the scope of the present study, another interesting avenue for future research would be using dynamic FNC to assess heterogeneous presentations of attention disorders such as ADHD.

Overall, our results add to accumulating evidence that we, as individuals, all fall somewhere on a spectrum ranging from highly attentive/motor-impulse controlled to highly inattentive/hyperactive-impulsive [74,75]. We show dynamic FNC analysis yields a process-focused understanding of large-scale neural connectivity and its association to attention problems and impulsivity. Our results in TD adolescents are consistent with clinical ADHD literature, underlying the importance of re-evaluating attention disorders like ADHD as being best conceptualized as extreme ends of a spectrum rather than categorically defined disorders.

## Supporting information

S1 Fig Static FNC results. Correlation matrix showing Static FNC results between ICs of Interest. dDMN = dorsal default mode network; pvDMN = posterior ventral default mode network; pdDMN = posterior dorsal default mode network; rECN = right executive control network; lECN = left executive control network; SN = salience network.
(DOCX)

S1 Table Independent sample t-tests between included and excluded participants. Independent sample T-tests revealed no significant differences between included and excluded participants for age nor behavioral measures of interest.
(DOCX)

S2 Table Spatial correlations results. Spatial correlation coefficients between ICs of interest with functional network templates (http://findlab.stanford.edu/functional_ROIs.html).
(DOCX)

S3 Table Correlations between dynamic states and framewise displacement. Pearson correlations coefficients between the mean framewise displacement and the occurrence of each dynamic FNC state per participant.
(DOCX)
