# Supplementary material for: The continuum of attention dysfunction: Evidence from dynamic functional network connectivity analysis in neurotypical adolescents
Source: PLoS One. 2023 Jan 20;18(1):e0279260. doi: 10.1371/journal.pone.0279260 (PMC9858399; doi:10.1371/journal.pone.0279260)
Supplement: S1 Fig — Correlation matrix showing Static FNC results between ICs of Interest. dDMN = dorsal default mode network; pvDMN = posterior ventral default mode network; pdDMN = posterior dorsal default mode network; rECN = right executive control network; lECN = left executive control network; SN = salience network. (DOCX) [file pone.0279260.s001.docx]

**Supporting Information**


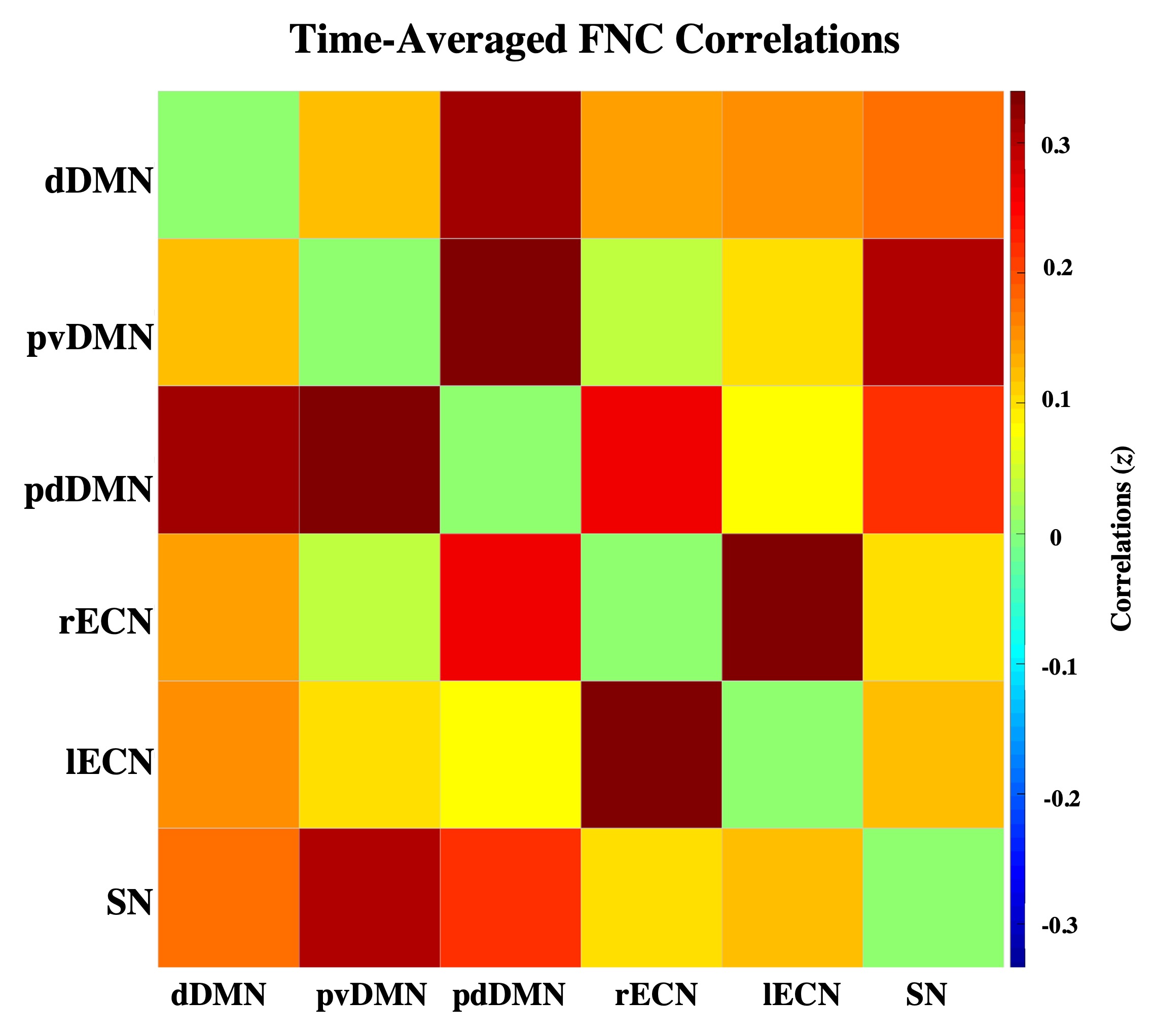


**S4 Figure. Static FNC Results.** Correlation matrix showing Static FNC results between ICs of Interest. dDMN = dorsal default mode network; pvDMN= posterior ventral default mode network; pdDMN = posterior dorsal default mode network; rECN = right executive control network; lECN = left executive control network; SN = salience network.
