# Supplementary material for: The continuum of attention dysfunction: Evidence from dynamic functional network connectivity analysis in neurotypical adolescents
Source: PLoS One. 2023 Jan 20;18(1):e0279260. doi: 10.1371/journal.pone.0279260 (PMC9858399; doi:10.1371/journal.pone.0279260)
Supplement: S1 Table — Independent sample T-tests revealed no significant differences between included and excluded participants for age nor behavioral measures of interest. (DOCX) [file pone.0279260.s002.docx]

**Supporting Information**

**S1 Table. Independent sample t-tests between included and excluded participants.** Independent sample T-tests revealed no significant differences between included and excluded participants for age nor behavioral measures of interest.

|  | t | df | Sig. | 95% Confidence Interval | |
| --- | --- | --- | --- | --- | --- |
|  |  |  |  | Lower | Upper |
| Age | 0.23 | 87 | 0.82 | -0.92 | 1.16 |
| IQ (WISC-IV, Cubes Standardized Score) | 0.737 | 87 | 0.46 | -1.44 | 3.14 |
| YSR Attention Problems | -1.66 | 87 | 0.10 | -8.96 | 0.79 |
| YSR ADHD | -0.75 | 87 | 0.45 | -5.71 | 2.57 |
| YSR Internalizing | -0.57 | 87 | 0.57 | -8.43 | 4.67 |
| YSR Externalizing | -0.65 | 87 | 0.52 | -7.55 | 3.82 |
| UPPS Urgency | 0.63 | 87 | 0.53 | -0.22 | 0.41 |
| UPPS Lack of Premeditation | -0.72 | 87 | 0.48 | -0.69 | 0.34 |
| UPPS Lack of Perseverance | -0.60 | 87 | 0.55 | -0.74 | 0.42 |
| UPPS Sensation Seeking | -0.34 | 87 | 0.73 | -0.63 | 0.46 |
