# Supplementary material for: The continuum of attention dysfunction: Evidence from dynamic functional network connectivity analysis in neurotypical adolescents
Source: PLoS One. 2023 Jan 20;18(1):e0279260. doi: 10.1371/journal.pone.0279260 (PMC9858399; doi:10.1371/journal.pone.0279260)
Supplement: S2 Table — Spatial correlation coefficients between ICs of interest with functional network templates (http://findlab.stanford.edu/functional_ROIs.html). (DOCX) [file pone.0279260.s003.docx]

**Supporting Information**

**S2 Table. Spatial correlations results.** Spatial correlation coefficients between ICs of interest with functional network templates (http://findlab.stanford.edu/functional_ROIs.html).

| Template | Most Correlated IC | Correlation Coefficient |
| --- | --- | --- |
| dDMN | 22 | 0.46 |
| pvDMN | 13 | 0.33 |
| pdDMN | 7 | 0.63 |
| rECN | 11 | 0.40 |
| lECN | 14 | 0.34 |
| SN | 27 | 0.35 |
