# Supplementary material for: The continuum of attention dysfunction: Evidence from dynamic functional network connectivity analysis in neurotypical adolescents
Source: PLoS One. 2023 Jan 20;18(1):e0279260. doi: 10.1371/journal.pone.0279260 (PMC9858399; doi:10.1371/journal.pone.0279260)
Supplement: S3 Table — Pearson correlations coefficients between the mean framewise displacement and the occurrence of each dynamic FNC state per participant. (DOCX) [file pone.0279260.s004.docx]

**Supporting Information**

**S3 Table.** **Correlations between dynamic states and framewise displacement.** Pearson correlations coefficients between the mean framewise displacement and the occurrence of each dynamic FNC state per participant.

|  | Coefficient | *p*-value |
| --- | --- | --- |
| State 1 | -0.1551717 | 0.16931917 |
| State 2 | -0.0033445 | 0.97651084 |
| State 3 | 0.15495473 | 0.16992346 |
| State 4 | 0.02312143 | 0.83868425 |
